# Three Years of the Coronavirus Disease 2019 Pandemic in a European Region: A Population-Based Longitudinal Assessment in Madrid Between 2020 and 2022

**DOI:** 10.1093/ofid/ofad635

**Published:** 2023-12-18

**Authors:** Juan Berenguer, María J Calvo-Alcántara, Alejandro Alvaro-Meca, José C Estévez, Miguel Basanta, Sergio Ruiz, Ángel L Matáix, César Bienzóbas, Lourdes Cosano, Aura P Silva, Pilar Salas, Pedro Gullón, Manuel Franco, José R Arribas, José M Molero, Miguel A Hernán

**Affiliations:** Infectious Diseases, Hospital General Universitario Gregorio Marañón, Madrid, Spain; Instituto de Investigación Sanitaria Gregorio Marañón, Madrid, Spain; Centro de Investigación Biomédica en Red de Enfermedades Infecciosas, Madrid, Spain; Subdirección General de Farmacia y Productos Sanitarios, Madrid, Spain; Centro de Investigación Biomédica en Red de Enfermedades Infecciosas, Madrid, Spain; Medicina Preventiva y Salud Pública, Universidad Rey Juan Carlos, Madrid, Spain; Gerencia Asistencial de Atención Primaria, Madrid, Spain; Dirección General de Sistemas de Información y Equipamientos Sanitarios, Madrid, Spain; Gerencia Asistencial de Atención Primaria, Madrid, Spain; Subdirección General de Farmacia y Productos Sanitarios, Madrid, Spain; Dirección General de Inspección y Ordenación Sanitaria, Madrid, Spain; Infectious Diseases, Hospital General Universitario Gregorio Marañón, Madrid, Spain; Infectious Diseases, Hospital General Universitario Gregorio Marañón, Madrid, Spain; Infectious Diseases, Hospital General Universitario Gregorio Marañón, Madrid, Spain; Departamento de Cirugía, Ciencias Médicas y Sociales, Grupo de Investigación en Epidemiología y Salud Pública, Universidad de Alcalá, Alcalá de Henares, Madrid, Spain; Departamento de Cirugía, Ciencias Médicas y Sociales, Grupo de Investigación en Epidemiología y Salud Pública, Universidad de Alcalá, Alcalá de Henares, Madrid, Spain; Centro de Investigación Biomédica en Red de Enfermedades Infecciosas, Madrid, Spain; Infectious Diseases, Internal Medicine, Hospital Universitario La Paz, Madrid, Spain; Instituto de Investigación Hospital Universitario La Paz, Madrid, Spain; Universidad Autónoma de Madrid, Madrid, Spain; Centro de Salud San Andrés, Madrid, Spain; CAUSALab, Departments of Epidemiology and Biostatistics, Harvard T. H. Chan School of Public Health, Boston, Massachusetts, USA

**Keywords:** coronavirus, COVID-19, population-based study, SARS-CoV-2

## Abstract

**Background:**

Our objective was to assess the health impact of coronavirus disease 2019 (COVID-19) during 2020–2022 in the Madrid region.

**Methods:**

We included all individuals registered in the Madrid Health System Registry as of 31 December 2019, and followed them until 31 December 2022. Using a unique personal identifier, we linked the databases of primary care, hospitals, pharmacies, certified laboratories performing diagnostic tests, vaccines, and mortality.

**Results:**

Of 6 833 423 individuals, 21.4% had a confirmed COVID-19 diagnosis, and 1.5% had a COVID-19 hospitalization (primary diagnosis). Thirty-day mortality was 1.6% for confirmed COVID-19 (from 11.4% in first semester 2020 to 0.4% in first semester 2022). Thirty-day mortality was 10.8% for COVID-19 hospitalizations (from 14.0% in first semester 2020 to 6.0% in second semester 2022). There were 24 073 deaths within 30 days of a confirmed COVID-19 diagnosis. Advanced age, male sex, higher socioeconomic deprivation, and comorbidities were associated with higher mortality.

**Conclusions:**

By linking administrative and clinical databases, we characterized the burden of the COVID-19 pandemic in Madrid over 3 years. Our analysis proposes a high-level framework for comparisons of the burden of COVID-19 across areas worldwide.

## Teaser

Using population-based data from several linked administrative and clinical databases, we characterized the health burden of COVID-19 longitudinally in the nearly 7 million residents of the Madrid region over the first 3 years and 7 epidemic waves of the pandemic.

The coronavirus disease 2019 (COVID-19) pandemic is among history's most widely studied public health crises. Many studies have described multiple aspects of the epidemiology of COVID-19, including its incidence, morbidity, mortality, and risk factors [1–4]. However, few integrated descriptions exist of the epidemiologic evolution and health consequences of the pandemic in entire populations for extended periods [5]. Longitudinal studies of COVID-19 at the population level are needed to characterize the pandemic's impact and to compare it across locations [6].

The most helpful population-level studies of COVID-19 would (1) cover the entire population in a particular geographic area; (2) encompass both the pre- and postvaccination phases of the pandemic as well as the spread of different variants of concern of severe acute respiratory syndrome coronavirus 2 (SARS-CoV-2); and (3) be based on high-quality data sources that can be used to link data for each individual in the population and thus allow integration of data on documented infections, hospitalizations, admissions to intensive care unit (ICU), deaths, and vaccinations with clinical and socioeconomic information. The availability of high-resolution clinical data is critical. For example, most studies have been unable to distinguish between hospitalizations with COVID-19 coded as a primary diagnosis versus secondary diagnosis [5, 7–10], which precludes an accurate quantification of the impact on the disease, both overall and by age group.

Here, we provide an integrated summary of the health impact of COVID-19 in 2020–2022 in the Madrid region, a population with universal access to healthcare that, as of June 2018, had the highest life expectancy in the European Union [11]. By linking information from several population registries and databases, we characterized the cohort of 6.8 million Madrid residents in the period 2020–2022. Our longitudinal analysis proposes a high-level framework for comparisons of the burden of COVID-19 across metropolitan areas worldwide.

## METHODS

The Madrid region (Spanish: Comunidad de Madrid), located in the center of the Iberian Peninsula, is 1 of the 17 autonomous communities of Spain, which includes the second-largest metropolitan area in the European Union (Supplementary Figure 1). About twice the size of Rhode Island, it has a population of about 7 million people, half of whom are within the city limits of Madrid. The Madrilenian Health Service (Servicio Madrileño de Salud [SERMAS]) provides healthcare services and public health programs in the Madrid region.

Our study includes all residents of Madrid on 31 December 2019, and summarizes their COVID-19–related data through 31 December 2022. To do so, we linked individual-level data from several population registries managed by SERMAS.

### Data Sources

#### Madrid Health System Registry

We included all individuals registered in the Madrid Health System Registry (SIP-CIBELES) as of 31 December 2019. Since healthcare coverage is universal in Madrid, this registry includes virtually the entire population (99.9% of individuals included in municipal registers). SIP-CIBELES also contains data on temporary residents coming from other Spanish regions or European Union member states, undocumented immigrants, and special populations (asylum seekers and victims of human trafficking). Among other variables, the registry includes the date of birth, sex, district of residence, date of death, and a unique personal identification code (Código de Identificación Personal Autonómico [CIPA]) for linkage with other databases [12].

We computed a previously proposed socioeconomic deprivation index using 6 census tract indicators related to occupation, unemployment, education, and internet access [13]. This deprivation index has been found to be associated with higher mortality [14] and COVID-19 incidence up to September 2021 [15]. Since we only had access to the resident's postal code, we averaged the census tract values (weighted by total population) in each postal code.

#### Primary Care Database

The electronic health records database for primary care (AP-Madrid) includes data on all interactions of Madrid residents with the network of primary care centers. AP-Madrid is linked to all public and certified private laboratories with molecular or antigen diagnostic test results for COVID-19. AP-Madrid is also linked to the vaccine registry with data on the types and dates of COVID-19 vaccines. Patient data and clinical activity are based on the latest International Classification of Primary Care (ICPC-2) [16]. We extracted diagnoses of obesity, diabetes mellitus, hypertension, cardiovascular disease (ischemic heart disease, heart failure, atrial fibrillation), cerebrovascular disease (stroke, transient ischemic attack), asthma, chronic obstructive pulmonary disease (COPD), chronic renal failure, connective tissue disease (rheumatoid arthritis, ankylosing spondylitis, systemic lupus erythematosus, other), liver cirrhosis, solid and hematologic neoplasms (diagnosed in the previous 5 years), human immunodeficiency virus (HIV) infection, organ transplantation, depressive disorder, psychosis (affective psychosis, schizophrenia, other organic psychoses), and dementia (see ICPC-2 codes listed in Supplementary Annex 1).

#### Hospital Database

The Minimum Basic Data Set (Conjunto Mínimo Básico de Datos) records all admissions to hospitals (public, private, public/private) that provide publicly funded healthcare in the region. Clinical documentation specialists at each institution code the information at discharge according to standardized procedures using the *International Classification of Diseases, 10th Revision, Clinical Modification* [*ICD-10-CM*]) [17]. The database includes demographics, dates of admission and discharge, hospital wards during admission (including ICU), comorbidities at entry, procedures during hospitalization, primary and secondary diagnoses at discharge and whether they were present on admission, and vital status at discharge (Supplementary Annex 2). Charlson comorbidities present at admission were determined using the *ICD-10* coding algorithms developed by Quan et al [18] (Supplementary Annex 3).

#### Pharmacy Database

Farmadrid registers all prescription drugs dispensed at community and hospital pharmacies in Madrid; medications are coded using the Anatomical Therapeutic Chemical classification system [19].

#### Viral Variants of Concern

Information about the SARS-CoV-2 variants of concern was obtained from the Weekly Epidemiological Report from the Epidemiological Surveillance Network of the Community of Madrid [20]. This bulletin summarizes the screening data of viral variants by specific polymerase chain reaction (PCR) provided by the reference microbiology laboratories. Given the excellent correlation between screening by specific PCR and the result by sequencing, this information provides a valid indicator of the circulation of viral variants in the region. The weekly distribution of viral variants during 2021 and 2022 in Madrid, the week in which they were first detected, and the week in which they became predominant (>50% of those screened) are shown in Supplementary Figure 2.

### Outcomes

We studied confirmed COVID-19, COVID-19 hospitalization, admission to ICU, need for mechanical ventilation, and length of hospital stays. We also assessed death within 30 days of a positive diagnostic test for SARS-CoV-2 and death within 30 days from the hospital admission date for COVID-19 hospitalizations.

Confirmed COVID-19 was defined as either a positive PCR or antigen test for SARS-CoV-2. The primary care database included all laboratory-confirmed COVID-19 cases up to December 2021. Since January 2022, antigen tests are available without a prescription, and their results have not been uniformly reported to the health system. From 1 July 2020 to 1 June 2022, a PCR for SARS-CoV-2 was routinely performed upon hospital admission to all patients, irrespective of symptomatology. Since June 2022, diagnostic tests were performed at the time of admission if symptoms suggestive of COVID-19 were present.

COVID-19 hospitalization was defined as a hospitalization with COVID-19 present on admission with an *ICD-10-CM* code of COVID-19 as a primary or secondary diagnosis. Hospital-acquired COVID-19 was defined as a COVID-19 infection not present on admission in which the positive diagnostic test was performed between days 8–14 (probable) or after day 14 (definite) after admission [21].

### Data Analysis

We used descriptive statistics (proportions, median and interquartile range [IQR]) to describe the sociodemographic and clinical characteristics of the study population. We computed the cumulative incidence (incidence proportion) of mortality after a documented COVID-19 diagnosis and after COVID-19 hospitalization. All calculations were made overall and for each of the 6 semesters (ie, 6-month periods) in the period 2020–2022, restricted to individuals alive at the beginning of each semester. Analyses were performed with Phyton (version 3.9; Phyton Software Foundation, Beaverton, Oregon).

### Ethics Approval

Ethics approval was provided by the institutional review board of the first author's institution (code: FIB-ANA-2020-08).

## RESULTS

The study cohort included 6 833 423 residents in Madrid on 31 December 2019. Table 1 summarizes their characteristics and selected comorbidities. Their median age was 42 (IQR, 25–58) years, 52.0% were female (see age pyramid by sex in Supplementary Figure 3), and 4.5% were potentially immunosuppressed (including those with diagnoses of cancer, connective tissue disease, organ transplantation, and HIV infection). Among adults aged ≥50 years, the most frequently recorded comorbidities were hypertension (41.2%), obesity (14.8%), diabetes mellitus type 2 (15.1%), depression (13.6%), and chronic heart disease (12.1%) (Supplementary Table 1). Among children (aged <18 years), asthma (11.2%) was the most frequent comorbidity.

**Table 1.** Baseline Characteristics of Residents in the Madrid Region on 31 December 2019

Characteristic	All	Age Group						
		0–17 y	18–39 y	40–49 y	50–59 y	60–69 y	70–79 y	≥80 y
Total No.	6 833 423	1 239 900	1 828 873	1 232 837	984 011	681 755	505 107	360 940
Age, y, median (IQR)	42 (24–58)	9 (5–13)	30 (24–35)	44 (42–47)	54 (52–57)	64 (62–67)	74 (72–76)	85 (82–89)
Male sex	3 278 367 (48.0)	636 602 (51.3)	889 621 (48.6)	610 902 (49.6)	477 386 (48.5)	314 084 (46.1)	222 273 (44.0)	127 499 (35.3)
History of comorbidities								
Hypertension	1 169 332 (17.1)	1227 (0.1)	27 750 (1.5)	97 243 (7.9)	208 519 (21.2)	270 507 (39.7)	301 953 (59.8)	262 133 (72.6)
Obesity	587 674 (8.6)	34 427 (2.8)	83 926 (4.6)	93 969 (7.6)	111 898 (11.4)	102 321 (15.0)	94 522 (18.7)	66 611 (18.5)
Depression	518 604 (7.6)	7163 (0.6)	76 398 (4.2)	91 199 (7.4)	103 228 (10.5)	91 283 (13.4)	80 230 (15.9)	69 103 (19.1)
Asthma	516 133 (7.6)	144 267 (11.6)	153 114 (8.4)	74 609 (6.1)	55 693 (5.7)	36 324 (5.3)	30 464 (6.0)	21 662 (6.0)
Diabetes	432 174 (6.3)	3013 (0.2)	14 803 (0.8)	30 916 (2.5)	71 633 (7.3)	103 987 (15.3)	116 482 (23.1)	91 340 (25.3)
Chronic heart disease^a^	322 349 (4.7)	1149 (0.1)	3636 (0.2)	11 011 (0.9)	32 593 (3.3)	60 243 (8.8)	92 923 (18.4)	120 794 (33.5)
Solid tumor	151 571 (2.2)	872 (0.1)	9276 (0.5)	15 885 (1.3)	25 469 (2.6)	33 981 (5.0)	37 619 (7.4)	28 469 (7.9)
Cerebrovascular disease^b^	146 966 (2.2)	1627 (0.1)	3847 (0.2)	7383 (0.6)	16 051 (1.6)	26 158 (3.8)	39 765 (7.9)	52 135 (14.4)
Connective tissue disease^c^	131 970 (1.9)	4288 (0.3)	17 829 (1.0)	21 417 (1.7)	27 794 (2.8)	24 769 (3.6)	20 761 (4.1)	15 112 (4.2)
COPD	108 746 (1.6)	230 (0.0)	808 (0.0)	3525 (0.3)	15 683 (1.6)	29 798 (4.4)	33 462 (6.6)	25 240 (7.0)
Chronic kidney disease	80 144 (1.2)	155 (0.0)	988 (0.1)	2064 (0.2)	5153 (0.5)	11 453 (1.7)	24 524 (4.9)	35 807 (9.9)
Dementia	75 153 (1.1)	137 (0.0)	283 (0.0)	467 (0.0)	1405 (0.1)	4155 (0.6)	18 395 (3.6)	50 311 (13.9)
Psychosis^d^	41 730 (0.6)	1211 (0.1)	8903 (0.5)	9323 (0.8)	9245 (0.9)	6284 (0.9)	4061 (0.8)	2703 (0.7)
HIV infection	26 873 (0.4)	459 (0.0)	7066 (0.4)	6884 (0.6)	9110 (0.9)	2593 (0.4)	634 (0.1)	127 (0.0)
Hematologic tumor	11 224 (0.2)	475 (0.0)	1227 (0.1)	1214 (0.1)	1696 (0.2)	2170 (0.3)	2508 (0.5)	1934 (0.5)
Cirrhosis	8979 (0.1)	28 (0.0)	204 (0.0)	856 (0.1)	2481 (0.3)	2605 (0.4)	1866 (0.4)	939 (0.3)
Solid organ transplant	2945 (0.0)	112 (0.0)	359 (0.0)	425 (0.0)	679 (0.1)	748 (0.1)	460 (0.1)	162 (0.0)

Data are presented as No. (%) unless otherwise indicated.

Abbreviations: COPD, chronic obstructive pulmonary disease; HIV, human immunodeficiency virus; IQR, interquartile range.

^a^Ischemic heart disease, heart failure, atrial fibrillation.

^b^Stroke, transient ischemic attack.

^c^Rheumatoid arthritis, ankylosing spondylitis, systemic lupus erythematosus, and other connective tissue disorders.

^d^Affective psychosis, schizophrenia, other organic psychoses.

### Confirmed COVID-19 Diagnoses and COVID-19 Hospitalizations

In the period 2020–2022, there were 1 667 787 confirmed COVID-19 diagnoses in 1 459 970 individuals (21.4% of the population), 96 941 hospitalizations (7.4% of all hospitalizations) with COVID-19 as the primary diagnosis in 90 704 individuals (1.3% of the population), and 7673 admissions to the ICU. Of these 96 941 hospitalizations, 47 934 occurred in 44 548 individuals aged ≥70 years, which is 8.8% of the population in that age group.

**Figure 1.**
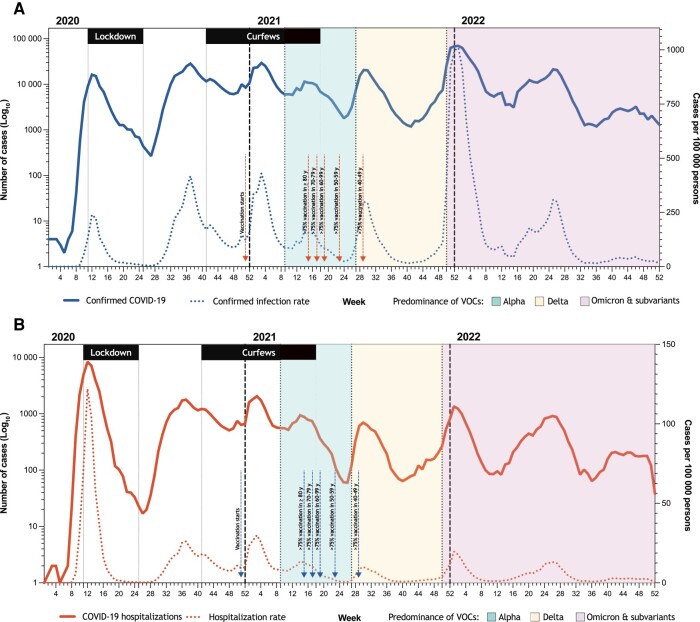
The graphs show the absolute number and rates per 100 000 persons of confirmed coronavirus disease 2019 (COVID-19) (*A*) and hospitalizations with COVID-19 as the primary diagnosis (*B*) over time. The graphs also show periods of lockdown and curfews, periods of the predominance of the different viral variants, and milestones of COVID-19 vaccination. Abbreviations: COVID-19, coronavirus disease 2019; VOCs, variants of concern.

Figure 1 shows the frequency of confirmed COVID-19 diagnoses and hospitalizations with COVID-19 as the primary diagnosis over time, as well as periods of lockdown and curfews, periods of the predominance of the different viral variants, and milestones of COVID-19 vaccination. The peak of 1008 confirmed COVID-19 per 100 000 persons occurred in week 1, 2022, 4 weeks after the Omicron variant was first detected in the region. The peak of 122 hospitalizations per 100 000 persons occurred during the first wave (week 12, 2020).

Table 2 shows the characteristics of individuals (56% male) hospitalized with COVID-19 as the primary diagnosis in 2020–2022. In the first semester of 2020, the median age at admission was 68, the Charlson comorbidity score was 1.0, and admission to ICU and mechanical ventilation was required in 5.9% and 6.2% of hospitalizations, respectively. In the second semester of 2022, the median age was 79, and the Charlson score 2.0, but ICU and mechanical ventilation decreased to 2.7% and 1.2%, respectively.

**Table 2.** Individuals Hospitalized With Coronavirus Disease 2019 as the Primary Diagnosis, Madrid Region, 2020–2022

Characteristic	2020		2021		2022		2020–2022
	1st Semester	2nd Semester	1st Semester	2nd Semester	1st Semester	2nd Semester	
Persons alive at the beginning of the period	6 833 522	6 787 716	6 762 325	6 736 679	6 714 698	6 708 682	6 833 522
No. of hospitalizations (per 100 000 persons)	32 347 (473.4)	20 519 (302.3)	19 035 (281.5)	6889 (102.3)	14 428 (214.9)	3723 (55.5)	96 941 (1418.6)
Age, y, median (IQR)	68.0 (55.0–79.0)	65.0 (52.0–79.0)	65.0 (53.0–76.0)	67.0 (50.0–83.0)	79.0 (68.0–86.0)	79.0 (69.0–85.0)	69.0 (55.0–81.0)
Male sex	18 383 (56.8)	11 578 (56.4)	11 045 (58.0)	3800 (55.2)	7750 (53.7)	1983 (53.3)	54 539 (56.3)
Charlson score on admission, median (IQR)	1.0 (0.0–2.0)	1.0 (0.0–2.0)	1.0 (0.0–2.0)	1.0 (0.0–3.0)	2.0 (1.0–4.0)	2.0 (1.0–4.0)	1.0 (0.0–2.0)
Charlson comorbidities on admission^a^							
Myocardial infarction	1162 (3.6)	687 (3.3)	684 (3.6)	295 (4.3)	709 (6.1)	401 (6.1)	3938 (4.1)
Congestive heart failure	2083 (6.4)	1593 (7.8)	1298 (6.8)	760 (11.0)	2326 (20.0)	1418 (21.7)	9478 (9.8)
Peripheral vascular disease	1007 (3.1)	693 (3.4)	673 (3.5)	296 (4.3)	849 (7.3)	438 (6.7)	3956 (4.1)
Cerebrovascular disease	1086 (3.4)	740 (3.6)	756 (4.0)	329 (4.8)	901 (7.8)	519 (8.0)	4331 (4.5)
Dementia	1679 (5.2)	1026 (5.0)	716 (3.8)	483 (7.0)	1577 (13.6)	921 (14.1)	6402 (6.6)
Chronic pulmonary disease	4720 (14.6)	3242 (15.8)	3217 (16.9)	1345 (19.5)	3263 (28.1)	2079 (31.8)	17 866 (18.4)
Rheumatic disease	713 (2.2)	486 (2.4)	433 (2.3)	190 (2.8)	531 (4.6)	285 (4.4)	2638 (2.7)
Peptic ulcer	116 (0.4)	85 (0.4)	63 (0.3)	34 (0.5)	63 (0.5)	48 (0.7)	409 (0.4)
Diabetes without chronic complication	6127 (18.9)	4259 (20.8)	3940 (20.7)	1482 (21.5)	2762 (23.8)	1643 (25.2)	20 213 (20.9)
Diabetes with chronic complication	963 (3.0)	738 (3.6)	714 (3.8)	372 (5.4)	888 (7.6)	507 (7.8)	4182 (4.3)
Hemiplegia or paraplegia	142 (0.4)	74 (0.4)	69 (0.4)	32 (0.5)	88 (0.8)	49 (0.8)	454 (0.5)
Renal disease	4485 (13.9)	2499 (12.2)	2419 (12.7)	1157 (16.8)	2528 (21.7)	1512 (23.2)	14 600 (15.1)
Liver disease, mild	1580 (4.9)	1271 (6.2)	1201 (6.3)	446 (6.5)	806 (6.9)	439 (6.7)	5743 (5.9)
Liver disease, moderate or severe	176 (0.5)	115 (0.6)	119 (0.6)	62 (0.9)	126 (1.1)	62 (0.9)	660 (0.7)
Any malignancy^b^	1469 (4.5)	846 (4.1)	819 (4.3)	468 (6.8)	1319 (11.3)	751 (11.5)	5672 (5.9)
Metastatic solid tumor	413 (1.3)	260 (1.3)	261 (1.4)	123 (1.8)	395 (3.4)	219 (3.4)	1671 (1.7)
HIV/AIDS	90 (0.3)	64 (0.3)	72 (0.4)	33 (0.5)	28 (0.2)	31 (0.5)	318 (0.3)
Admission to ICU	1903 (5.9)	1883 (9.2)	2294 (12.1)	902 (13.1)	592 (4.1)	99 (2.7)	7673 (7.9)
Mechanical ventilation	2019 (6.2)	1384 (6.7)	1864 (9.8)	670 (9.7)	371 (2.6)	44 (1.2)	6352 (6.6)
Length of stay, d, median (IQR)	8.0 (4.0–14.0)	8.0 (5.0–13.0)	8.0 (5.0–13.0)	7.0 (4.0–13.0)	6.0 (3.0–10.0)	5.0 (3.0–9.0)	7.0 (4.0–13.0)
Death within 30 d from admission							
All hospitalizations	4536 (14.0)	1791 (8.7)	1662 (8.7)	816 (11.8)	1429 (9.9)	224 (6.0)	10 458 (10.8)
ICU admission	498 (26.2)	232 (12.3)	251 (10.9)	110 (12.2)	94 (15.9)	19 (19.2)	1204 (15.7)
Mechanical ventilation	561 (27.8)	217 (15.7)	244 (13.1)	91 (13.6)	77 (20.8)	15 (34.1)	1205 (19.0)

Data are presented as No. (%) unless otherwise indicated.

Abbreviations: HIV, human immunodeficiency virus; ICU, intensive care unit; IQR, interquartile range.

^a^See *International Classification of Diseases, Tenth Revision* coding algorithms for Charlson comorbidities in Supplementary Appendix 3.

^b^Including lymphoma and leukemia, except malignant skin cancer.

The proportion of hospitalizations with COVID-19 as a secondary diagnosis increased from <10% in 2020 to 30% in 2022 (Supplementary Figure 4). Compared with patients hospitalized with COVID-19 as a primary diagnosis, these patients were younger, more likely to be female, and had a lower proportion of ICU admissions and mechanical ventilation (Supplementary Table 2).

The proportion of hospitalizations with probable or definite hospital-acquired COVID-19 was highest (5.3%) in the last semester of 2022 (Supplementary Figure 4). Compared to patients with hospitalizations with COVID-19 as a primary diagnosis, these patients were, on average, 3 years older, less likely to be male (43.4% vs 56.3%), had higher Charlson comorbidity scores, and had longer hospital stays (28 vs 7 days) (Supplementary Table 2).

### Vaccination

The COVID-19 vaccination rollout started in week 52, 2020, with national prioritization policies. In the period 2020–2022, 19 941 946 vaccine doses were administered. By December 2021, 75.2% of the population and 88.9% of those aged 50 or older had received at least 1 vaccine dose. The cumulative uptake of the vaccine in the study population overall and by age group is shown in Supplementary Figure 5. The vaccination status of the study population, including the proportion of those with 1, 2, and 3 doses, is shown in Supplementary Table 3.

### Antivirals

From April to December 2022, a total of 2937 COVID-19 ambulatory infections were treated with nirmatrelvir plus ritonavir and 115 with molnupiravir. These antivirals were authorized for use within the first 5 days of symptoms among ambulatory individuals with confirmed COVID-19 and severe immunosuppression or those aged ≥65 years with severe comorbidities (Supplementary Figure 6).

### Mortality

In 2020–2022, 30-day mortality was documented in 24 073 of the 1 459 970 (1.6%) confirmed COVID-19 diagnoses, with 20 241 deaths occurring in 191 683 (10.6%) diagnoses among people aged ≥70 years. The 30-day mortality risk after confirmed COVID-19 by sex, age, deprivation index, and comorbidities broken down by semesters is shown in Table 3. Mortality among confirmed cases was greatest in the first semester of 2020 (11.4%) and lowest in the first semester of 2022 (0.4%). Across the study period, mortality was higher for males than females and increased with age. According to the area-level deprivation index, mortality was 1.3% in the least deprived quintile and 1.9% in the most deprived quintile. Overall, people with dementia, COPD, chronic kidney disease, cerebrovascular disease, and chronic heart disease had a 30-day mortality risk of >10%.

**Table 3.** Thirty-Day Mortality Risk After Confirmed Coronavirus Disease 2019 Diagnosis by Period, Sex, Age, Deprivation Index, and Comorbidities, Madrid Region, 2020–2022

Characteristic	2020		2021		2022		2020–2022
	1st Semester	2nd Semester	1st Semester	2nd Semester	1st Semester	2nd Semester	
All	8726/76 282 (11.4)	4984/288 964 (1.7)	5047/256 614 (2.0)	2130/304 974 (0.7)	1995/447 233 (0.4)	1191/85 903 (1.4)	24 073/1 459 970 (1.6)
Sex							
Male	5172/34 748 (14.9)	2822/135 702 (2.1)	2954/122 881 (2.4)	1147/141 731 (0.8)	1033/186 275 (0.6)	606/33 524 (1.8)	13 734/654 861 (2.1)
Female	3554/41 534 (8.6)	2162/153 262 (1.4)	2093/133 733 (1.6)	983/163 243 (0.6)	962/260 958 (0.4)	585/52 379 (1.1)	10 339/805 109 (1.3)
Age group, y							
0–17	1/646 (0.2)	0/482 780	0/456 820	1/63 554 (0.0)	6/68 077 (0.0)	0/21 780	8/228 415 (0.0)
18–39	26/12 109 (0.2)	20/93 911 (0.0)	19/78 351 (0.0)	11/113 913 (0.0)	7/122 518 (0.0)	5/12 098 (0.0)	88/432 900 (0.0)
40–49	124/12 055 (1.0)	64/53 115 (0.1)	61/48 233 (0.1)	15/55 896 (0.0)	36/85 251 (0.0)	20/9891 (0.2)	320/264 441 (0.1)
50–59	350/14 615 (2.4)	230/41 731 (0.6)	217/39 140 (0.6)	108/35 933 (0.3)	92/60 366 (0.2)	51/13 822 (0.4)	1048/205 607 (0.5)
60–69	850/11 616 (7.3)	450/23 482 (1.9)	559/22 375 (2.5)	208/19 001 (1.1)	190/43 780 (0.4)	111/16 670 (0.7)	2368/136 924 (1.7)
70–79	2304/11 069 (20.8)	1050/14 683 (7.2)	1345/13 655 (9.8)	442/9455 (4.7)	424/38 550 (1.1)	255/18 013 (1.4)	5820/105 425 (5.5)
≥80	5071/14 172 (35.8)	3170/13 764 (23.0)	2846/9178 (31.0)	1345/7222 (18.6)	1240/28 691 (4.3)	749/13 231 (5.7)	14 421/86 258 (16.7)
Area-level deprivation index							
Quintile 1 (least deprived)	1432/14 778 (9.7)	669/49 819 (1.3)	772/50 482 (1.5)	271/59 980 (0.5)	300/85 693 (0.4)	179/15 715 (1.1)	3623/276 467 (1.3)
Quintile 2	1717/14 956 (11.5)	922/52 370 (1.8)	987/51 298 (1.9)	477/60 537 (0.8)	385/88 576 (0.4)	257/17 204 (1.5)	4745/284 941 (1.7)
Quintile 3	1751/15 908 (11.0)	1019/56 795 (1.8)	1038/54 684 (1.9)	458/64 170 (0.7)	426/95 348 (0.4)	218/17 606 (1.2)	4910/304 511 (1.6)
Quintile 4	1842/14 825 (12.4)	1187/62 202 (1.9)	1141/50 307 (2.3)	453/59 523 (0.8)	418/88 214 (0.5)	240/17 761 (1.4)	5281/292 832 (1.8)
Quintile 5 (most deprived)	1951/15 446 (12.6)	1168/66 268 (1.8)	1095/48 346 (2.3)	463/58 811 (0.8)	459/86 789 (0.5)	291/17 185 (1.7)	5427/292 845 (1.9)
History of comorbidities							
Hypertension	5770/27 264 (21.2)	3485/47 106 (7.4)	3401/40 785 (8.3)	1484/34 335 (4.3)	1319/87 185 (1.5)	783/33 510 (2.3)	16 242/270 185 (6.0)
Obesity	1959/12 023 (16.3)	1089/30 294 (3.6)	1130/25 507 (4.4)	525/24 667 (2.1)	419/45 928 (0.9)	223/13 111 (1.7)	5345/151 530 (3.5)
Depression	1563/10 376 (15.1)	901/22 469 (4.0)	881/19 747 (4.5)	459/21 265 (2.2)	391/43 745 (0.9)	235/13 180 (1.8)	4430/130 782 (3.4)
Asthma	469/5633 (8.3)	280/25 284 (1.1)	285/23 273 (1.2)	152/30 207 (0.5)	109/40 977 (0.3)	75/6979 (1.1)	1370/132 353 (1.0)
Diabetes	2744/11 693 (23.5)	1650/19 176 (8.6)	1708/16 091 (10.6)	707/13 186 (5.4)	599/31 953 (1.9)	339/12 317 (2.8)	7747/104 416 (7.4)
Chronic heart disease	2984/10 121 (29.5)	1885/13 248 (14.2)	1961/11 211 (17.5)	937/9062 (10.3)	801/27 355 (2.9)	459/11 714 (3.9)	9027/82 711 (10.9)
Solid tumor	939/3794 (24.7)	602/6087 (9.9)	576/5321 (10.8)	217/4596 (4.7)	204/11 789 (1.7)	119/4340 (2.7)	2657/35 927 (7.4)
Cerebrovascular disease	1288/4543 (28.4)	857/5939 (14.4)	772/5006 (15.4)	384/4271 (9.0)	331/12 716 (2.6)	217/5080 (4.3)	3849/37 555 (10.2)
Connective tissue disease	340/2566 (13.3)	223/6150 (3.6)	236/5527 (4.3)	101/5545 (1.8)	108/11 869 (0.9)	40/3335 (1.2)	1048/34 992 (3.0)
COPD	981/3335 (29.4)	679/4314 (15.7)	720/3755 (19.2)	320/3058 (10.5)	248/8719 (2.8)	158/3949 (4.0)	3106/27 130 (11.4)
Chronic kidney disease	714/2628 (27.2)	480/3416 (14.1)	524/2675 (19.6)	266/2219 (12.0)	224/6884 (3.3)	148/3217 (4.6)	2356/21 039 (11.2)
Dementia	1064/3745 (28.4)	622/3508 (17.7)	558/2250 (24.8)	265/1833 (14.5)	280/7523 (3.7)	177/2740 (6.5)	2966/21 599 (13.7)
Psychosis	172/814 (21.1)	62/1453 (4.3)	50/1276 (3.9)	23/1456 (1.6)	27/2721 (1.0)	14/905 (1.5)	348/8625 (4.0)
HIV infection	22/365 (6.0)	16/1392 (1.1)	30/1042 (2.9)	8/1214 (0.7)	8/1547 (0.5)	5/380 (1.3)	89/5940 (1.5)
Hematologic tumor	118/418 (28.2)	87/489 (17.8)	98/439 (22.3)	25/370 (6.8)	36/988 (3.6)	18/377 (4.8)	382/3081 (12.4)
Cirrhosis	55/227 (24.2)	56/366 (15.3)	49/334 (14.7)	9/261 (3.4)	24/705 (3.4)	23/287 (8.0)	216/2180 (9.9)
Solid organ transplant	17/123 (13.8)	10/166 (6.0)	14/145 (9.7)	10/161 (6.2)	7/321 (2.2)	1/142 (0.7)	59/1058 (5.6)

Columns show No. of deaths within 30 days of COVID-19 diagnosis/No. of people with confirmed COVID-19 diagnosis (%).

Abbreviations: COPD, chronic obstructive pulmonary disease; HIV, human immunodeficiency virus.

**Figure 2.**
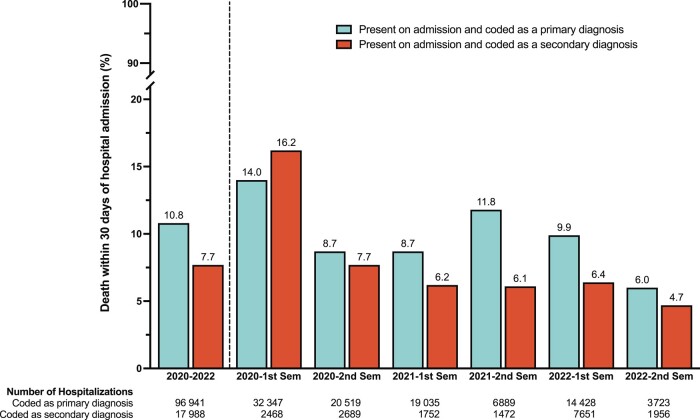
Thirty-day mortality from the date of hospital admission for coronavirus disease 2019 hospitalizations present on admission and coded as a primary (blue) or secondary (red) diagnosis. Abbreviation: Sem, semester (6-month period).

The 30-day mortality risk was 10.8% after hospitalization with COVID-19 as the primary diagnosis, ranging from 14.0% in the first semester of 2020 to 6.0% in the second semester of 2022 (Table 2). Mortality was higher among those needing ICU admission (15.7%) or mechanical ventilation (19.0%). The 30-day mortality risk was 7.7% for hospitalizations with COVID-19 coded as a secondary diagnosis, ranging from 16.2% in the first semester of 2020 to 4.7% in the second semester of 2022 (Figure 2 and Supplementary Table 4).

## DISCUSSION

We characterized the health burden of COVID-19 longitudinally over the first 3 years of the pandemic in the population of a large metropolitan area. By using population-based data from several linked administrative and clinical databases, we described the evolution of the pandemic in the nearly 7 million residents of Madrid over 3 years and 7 epidemic waves.

Our findings indicate that, between 2020 and 2022, 1.3% of the population in the region was hospitalized due to COVID-19, and 0.4% of the population died within 30 days from a COVID-19 diagnosis. Among people who were aged ≥70 years, nearly 9% required hospitalization due to COVID-19, and 2.3% died within 30 days from a COVID-19 diagnosis. The probability of dying from COVID-19 was greater in older persons, men, and those with comorbidities, especially dementia, COPD, chronic kidney disease, cerebrovascular disease, and chronic heart disease.

Like other countries in the European Union, Spain had a considerable reduction in life expectancy in 2020 [22]. However, life expectancy in the Madrid region continued to be among the highest in the European Union after the first 2 years of the pandemic [23].

A study like ours has been reported from the region of Stockholm, with complete population coverage of 1.8 million inhabitants and high-resolution data on patient characteristics and comorbid conditions [5]. Nevertheless, the study was limited to the first year of the pandemic and lacked information on vaccination rollout and frequency of viral variants.

A higher socioeconomic deprivation was associated with a smaller increase in mortality risk than in other studies in Europe [24]. Disease severity is also expected to have been affected by changes in the predominant viral variant [25–28], acquired immunity, and vaccination coverage [29–31] during each period. Of note, by December 2021, almost 75% of the population and almost 90% of those older than 50 years had received at least 1 dose of the vaccine.

About 20% of the population had a confirmed diagnosis of COVID-19 in the period 2020–2022. However, this figure is an underestimate of the proportion of infected individuals: A population-based seroprevalence study estimated that about 11.5% of the population of Madrid had already developed antibodies against SARS-CoV-2 by October 2020 [32]. The underestimation was particularly high during the first wave of the pandemic when, due to a lack of adequate testing, the number of hospitalizations was closest to the number of documented infections [33]. Beginning in the second semester of 2020, COVID-19 testing became widely available, and all individuals admitted to hospitals were systematically tested, which reduced the underestimation of infections. After December 2021, the underestimation of new diagnoses started to grow again because of the availability of home tests whose results were not systematically reported to the health system.

COVID-19 was the primary diagnosis in 7% of all hospitalizations during the study period, with 8% requiring ICU admission. The infection was present on admission but coded as a secondary diagnosis in 13% of hospitalizations with a COVID-19 code, reaching 30% when Omicron and its subvariants were predominant in 2022. Almost 2% of COVID-19 hospitalizations were hospital-acquired; these patients were older and sicker than those with community-acquired infections, a finding concordant with what has been found in other studies [34].

Individuals hospitalized with COVID-19 in 2022 had shorter stays and a lower risk of death, even though, on average, they were >10 years older and had more severe comorbidities than individuals hospitalized in the previous 2 years. Similar findings have also been reported in hospitals across the United States [35]. Of note, among those requiring critical care in 2022, mortality was similar to or higher than that in the pandemic's first semester, as has also been reported across hospitals in the United States [36].

Our study has the strengths of being population-based and with high-quality and integrated data sources. The most comparable previous study covered 1.8 million individuals from the Stockholm region [5] but was limited to the first year of the pandemic. Furthermore, our study could discriminate between the different types of COVID-19 hospitalizations, which is necessary (particularly during the Omicron dominance period) to distinguish hospitalizations due to COVID-19 from those with an incidental COVID-19 diagnosis. On the other hand, our study lacks individual data on race/ethnicity and socioeconomic status. Nonetheless, this limitation is partially compensated by data on a validated deprivation index at the postal code level. Limitations also include lack of information about smoking and alcohol use and on some pharmacological interventions that likely influenced the prognosis of COVID-19.

In conclusion, by linking population-based administrative and clinical databases, we characterized the health burden of the COVID-19 pandemic in Madrid over 3 years. Our longitudinal analysis proposes a high-level framework for comparisons of the burden of COVID-19 across areas worldwide. To better understand the impact of the COVID-19 pandemic on healthcare, these comparisons should include the direct morbidity and mortality from the disease, its postacute sequelae, and the disruptions it caused in routine healthcare.

## Supplementary Material

ofad635_Supplementary_Data

## Supplementary Data

Supplementary materials are available at *Open Forum Infectious Diseases* online. Consisting of data provided by the authors to benefit the reader, the posted materials are not copyedited and are the sole responsibility of the authors, so questions or comments should be addressed to the corresponding author.

## Notes

***Acknowledgments*.** This work uses data provided by patients and collected by professionals from SERMAS (Madrilenian Health System). We express our gratitude to Carlos Cevallos García, Marta Cortés García, Rafael Delgado Vázquez, María A. Martínez Huedo, Francisco L. Sánchez Prieto, José Verdejo Ortés, and Antonio Zapatero Gaviria for their support.

***Patient consent*.** Ethics approval was provided by the Ethics Committee for Research with Medicines of Hospital General Universitario Gregorio Marañón (code: FIB-ANA-2020-08). As the study utilized de-identified data, a waiver of informed consent was granted by this committee.
