# Supplementary material for: Three Years of the Coronavirus Disease 2019 Pandemic in a European Region: A Population-Based Longitudinal Assessment in Madrid Between 2020 and 2022
Source: Open Forum Infect Dis. 2023 Dec 18;11(1):ofad635. doi: 10.1093/ofid/ofad635 (PMC10763997; doi:10.1093/ofid/ofad635)
Supplement: ofad635_Supplementary_Data [file ofad635_supplementary_data.pdf]

Table S1. Characteristics of residents ≥ 50 years in the Madrid region on December 31, 2019

Characteristic	N=2531961
**Age, median (IQR), years**	63.0 (56.0; 74.0)
**Male sex, no. (%)**	1,141,324 (45.1)
**Comorbidities, no. (%)**	
Hypertension	1,043,112 (41.2)
Obesity	375,352 (14.8)
Diabetes	383,442 (15.1)
Depression	343,844 (13.6)
Chronic heart disease *	306,553 (12.1)
Asthma	144,143 (5.7)
Solid Tumor	125,538 (5.0)
Cerebrovascular disease †	134,109 (5.3)
COPD	104,183 (4.1)
Connective tissue disease ‡	88,436 (3.5)
Dementia	74,266 (2.9)
Chronic Renal Failure	76,937 (3.0)
Psychosis #	22,293 (0.9)
HIV infection	12,464 (0.5)
Hematologic Tumor	8,308 (0.3)
Cirrhosis	7,891 (0.3)
Transplant	2,049 (0.1)

* Ischemic heart disease, Heart failure, Atrial fibrillation
† Stroke, Transient ischemic attack
‡ Rheumatoid arthritis, Ankylosing spondylitis, Systemic lupus erythematosus, other connective tissue disorders
# Affective psychosis, Schizophrenia, other organic psychoses

Abbreviations: COPD, chronic obstructive pulmonary disease; HIV, Human immunodeficiency virus

Table S2. Characteristics of hospitalizations with a COVID-19 ICD-10-CM code in the Madrid region, 2020-2022.

	2020		2021		2022		2020-2022
	1st semester	2nd semester	1st semester	2nd semester	1st semester	2nd semester	
Alive at the beginning of the period)	6833522	6787716	6762325	6736679	6714698	6708682	6833522
**All hospitalizations with a COVID-19 ICD-10-CM code, N (%)**							
Number of hospitalizations	38896	27321	28854	12239	25452	6403	139165
Hospitalizations per 100,000 persons	569.2	402.5	426.7	181.7	379.0	95.4	2036.5
Age - years, median (IQR)	66.0 (53.0 - 79.0)	64.0 (50.0 - 79.0)	62.0 (50.0 - 75.0)	62.0 (43.0 - 79.0)	76.0 (61.0 - 85.0)	76.0 (64.0 - 84.0)	67.0 (52.0 - 80.0)
Male sex, N (%)	21638 (55.6)	14895 (54.5)	16370 (56.7)	6546 (53.5)	12913 (50.7)	3213 (50.2)	75575 (54.3)
Charlson score, median (IQR)	0.0 (0.0 - 2.0)	1.0 (0.0 - 2.0)	0.0 (0.0 - 2.0)	1.0 (0.0 - 2.0)	2.0 (0.0 - 4.0)	2.0 (1.0 - 4.0)	1.0 (0.0 - 2.0)
Admission to ICU, N (%)	2155 (5.5)	2375 (8.7)	2947 (10.2)	1364 (11.1)	1415 (5.6)	320 (5.0)	10576 (7.6)
Admission to ICU, per 100,000 persons	31.5	35.0	43.6	20.2	21.1	4.8	154.8
Mechanical ventilation, N (%)	2157 (5.5)	1689 (6.2)	2307 (8.0)	927 (7.6)	625 (2.5)	95 (1.5)	7800 (5.6)
Mechanical ventilation, per 100,000 persons	31.6	24.9	34.1	13.8	9.3	1.4	114.1
Length of stay - days, median (IQR)	7.0 (4.0 - 14.0)	8.0 (5.0 - 14.0)	7.0 (5.0 - 13.0)	7.0 (4.0 - 14.0)	6.0 (3.0 - 12.0)	6.0 (4.0 - 12.0)	7.0 (4.0 - 13.0)
**COVID-19 present on admission coded as primary diagnosis, N (%)**							
Number of hospitalizations	32347	20519	19035	6889	14428	3723	96941
Hospitalizations per 100,000 persons	473.4	302.3	281.5	102.3	214.9	55.5	1418.6
Age - years, median (IQR)	68.0 (55.0 - 79.0)	65.0 (52.0 - 79.0)	65.0 (53.0 - 76.0)	67.0 (50.0 - 83.0)	79.0 (68.0 - 86.0)	79.0 (69.0 - 85.0)	69.0 (55.0 - 81.0)
Male sex, N (%)	18383 (56.8)	11578 (56.4)	11045 (58.0)	3800 (55.2)	7750 (53.7)	1983 (53.3)	54539 (56.3)
Charlson score, median (IQR)	1.0 (0.0 - 2.0)	1.0 (0.0 - 2.0)	1.0 (0.0 - 2.0)	1.0 (0.0 - 3.0)	2.0 (1.0 - 4.0)	2.0 (1.0 - 4.0)	1.0 (0.0 - 2.0)
Admission to ICU, N (%)	1903 (5.9)	1883 (9.2)	2294 (12.1)	902 (13.1)	592 (4.1)	99 (2.7)	7673 (7.9)
Admission to ICU, per 100,000 persons	27.8	27.7	33.9	13.4	8.8	1.5	112.3
Mechanical ventilation, N (%)	2019 (6.2)	1384 (6.7)	1864 (9.8)	670 (9.7)	371 (2.6)	44 (1.2)	6352 (6.6)
Mechanical ventilation, per 100,000 persons	29.5	20.4	27.6	9.9	5.5	0.7	93.0
Length of stay - days, median (IQR)	8.0 (4.0 - 14.0)	8.0 (5.0 - 13.0)	8.0 (5.0 - 13.0)	7.0 (4.0 - 13.0)	6.0 (3.0 - 10.0)	5.0 (3.0 - 9.0)	7.0 (4.0 - 13.0)
**COVID-19 present on admission coded as secondary diagnosis, N (%)**							
Number of hospitalizations	2468	2689	1752	1472	7651	1956	17988
Hospitalizations per 100,000 persons	36.1	39.6	25.9	21.9	113.9	29.2	263.2
Age - years, median (IQR)	74.0 (56.0 - 85.0)	63.0 (36.0 - 80.0)	63.0 (38.0 - 79.0)	55.5 (32.0 - 77.0)	69.0 (43.0 - 82.0)	72.0 (54.0 - 82.0)	68.0 (43.0 - 82.0)
Male sex, N (%)	1136 (46.0)	1100 (40.9)	777 (44.3)	568 (38.6)	3343 (43.7)	890 (45.5)	7814 (43.4)
Charlson score, median (IQR)	1.0 (0.0 - 3.0)	1.0 (0.0 - 3.0)	1.0 (0.0 - 3.0)	1.0 (0.0 - 3.0)	2.0 (0.0 - 4.0)	2.0 (0.0 - 4.0)	1.0 (0.0 - 3.0)
Admission to ICU, N (%)	160 (6.5)	169 (6.3)	113 (6.4)	129 (8.8)	544 (7.1)	148 (7.6)	1263 (7.0)
Admission to ICU, per 100,000 persons	2.3	2.5	1.7	1.9	8.1	2.2	18.5
Mechanical ventilation, N (%)	82 (3.3)	74 (2.8)	46 (2.6)	49 (3.3)	147 (1.9)	30 (1.5)	428 (2.4)
Mechanical ventilation, per 100,000 persons	1.2	1.1	0.7	0.7	2.2	0.4	6.3

Length of stay - days, median (IQR)	8.0 (4.0 - 16.0)	6.0 (3.0 - 14.0)	6.0 (3.0 - 13.0)	6.0 (3.0 - 13.0)	6.0 (3.0 - 11.0)	6.5 (3.0 - 12.0)	6.0 (3.0 - 13.0)
**Hospital acquired COVID-19, N (%)**							
Number of hospitalizations	748	756	560	555	1082	340	4041
Hospitalizations per 100,000 persons	10.9	11.1	8.3	8.2	16.1	5.1	59.1
Age - years, median (IQR)	58.0 (47.0 - 71.0)	75.0 (62.0 - 83.0)	72.0 (58.0 - 82.2)	74.0 (61.0 - 84.0)	75.0 (64.0 - 83.0)	76.0 (66.0 - 83.0)	72.0 (58.0 - 82.0)
Male sex, N (%)	353 (47.2)	376 (49.7)	286 (51.1)	280 (50.5)	546 (50.5)	165 (48.5)	2006 (49.6)
Charlson score, median (IQR)	0.0 (0.0 - 2.0)	2.0 (1.0 - 4.0)	2.0 (0.8 - 4.0)	2.0 (1.0 - 4.0)	2.5 (1.0 - 4.0)	2.0 (1.0 - 4.0)	2.0 (0.0 - 4.0)
Admission to ICU, N (%)	45 (6.0)	76 (10.1)	62 (11.1)	69 (12.4)	132 (12.2)	33 (9.7)	417 (10.3)
Admission to ICU, per 100,000 persons	0.7	1.1	0.9	1.0	2.0	0.5	6.1
Mechanical ventilation, N (%)	33 (4.4)	61 (8.1)	42 (7.5)	40 (7.2)	57 (5.3)	8 (2.4)	241 (6.0)
Mechanical ventilation, per 100,000 persons	0.5	0.9	0.6	0.6	0.8	0.1	3.5
Length of stay - days, median (IQR)	10.0 (4.0 - 31.0)	37.0 (24.8 - 59.0)	27.0 (15.0 - 44.2)	35.0 (22.0 - 58.0)	29.0 (19.2 - 46.0)	28.0 (19.0 - 42.2)	28.0 (16.0 - 48.0)

Table S3. COVID-19 vaccination status in the Madrid region, 2020-2022

	2020		2021		2022		2020-2022
	1st semester	2nd semester	1st semester	2nd semester	1st semester	2nd semester	
**All the population**							
Persons alive at the beginning of the period	6,833,522	6,787,716	6,762,325	6,736,679	6,714,698	6,708,682	6,833,522
Persons with 1 vaccine dose (%)	15 (0.0)	3,276 (0.0)	3,071,164 (45.4)	5,064,533 (75.2)	5,221,943 (77.8)	5,223,937 (77.9)	5,256,973 (76.9)
Persons with 2 vaccine doses (%)	6 (0.0)	125 (0.0)	2,032,090 (30.1)	4,467,932 (66.3)	4,883,822 (72.7)	4,908,204 (73.2)	4,937,661 (72.3)
Persons with 3 vaccine doses (%)	-	-	554 (0.0)	1,401,046 (20.8)	2,689,284 (40.1)	2,873,553 (42.8)	2,881,326 (42.2)
**Individuals aged $\geq$ 50 years**							
Persons alive at the beginning of the period	2,531,961	2,487,471	2,462,935	2,438,221	2,417,037	2,411,286	2,531,961
Persons with 1 vaccine dose (%)	10 (0.0)	2,750 (0.1)	2,102,557 (85.4)	2,168,132 (88.9)	2,153,295 (89.1)	2,148,638 (89.1)	2,180,845 (86.1)
Persons with 2 vaccine doses (%)	6 (0.0)	60 (0.0)	1,649,150 (67.0)	2,078,443 (85.2)	2,112,822 (87.4)	2,110,852 (87.5)	2,139,655 (84.5)
Persons with 3 vaccine doses (%)	-	-	486 (0.0)	1,244,799 (51.1)	1,691,124 (70.0)	1,756,590 (72.8)	1,764,274 (69.7)

Table S4. Thirty-day mortality after hospital admission for hospitalizations with COVID-19, Madrid region, 2020-2022

	2020		2021		2022		2020-2022
	1st semester	2nd semester	1st semester	2nd semester	1st semester	2nd semester	
**All COVID-19 hospitalizations**							
All hospitalizations	5059/38896 (13.0)	2168/27321 (7.9)	2260/28854 (7.8)	927/12239 (7.6)	1706/25452 (6.7)	927/6403 (14.5)	13047/139165 (9.4)
With ICU admission	537/2155 (24.9)	316/2375 (13.3)	331/2947 (11.2)	149/1364 (10.9)	160/1415 (11.3)	42/320 (13.1)	1535/10576 (14.5)
With mech. ventilation	593/2157 (27.5)	287/1689 (17.0)	314/2307 (13.6)	123/927 (13.3)	117/625 (18.7)	26/95 (27.4)	1460/7800 (18.7)
**COVID-19 present on admission and coded as primary diagnosis**							
All hospitalizations	4536/32347 (14.0)	1791/20519 (8.7)	1662/19035 (8.7)	816/6889 (11.8)	1429/14428 (9.9)	224/3723 (6.0)	10458/96941 (10.8)
With ICU admission	498/1903 (26.2)	232/1883 (12.3)	251/2294 (10.9)	110/902 (12.2)	94/592 (15.9)	19/99 (19.2)	1204/7673 (15.7)
With mech. ventilation	561/2019 (27.8)	217/1384 (15.7)	244/1864 (13.1)	91/670 (13.6)	77/371 (20.8)	15/44 (34.1)	1205/6352 (19.0)
**COVID-19 present on admission and coded as secondary diagnosis**							
All hospitalizations	399/2468 (16.2)	208/2689 (7.7)	108/1752 (6.2)	90/1472 (6.1)	486/7651 (6.4)	91/1956 (4.7)	1382/17988 (7.7)
With ICU admission	26/160 (16.2)	26/169 (15.4)	13/113 (11.5)	14/129 (10.9)	51/544 (9.4)	13/148 (8.8)	143/1263 (11.3)
With mech. ventilation	20/82 (24.4)	20/74 (27.0)	11/46 (23.9)	11/49 (22.4)	32/147 (21.8)	8/30 (26.7)	102/428 (23.8)
**Hospital-associated COVID-19**							
All hospitalizations	78/748 (10.4)	158/756 (20.9)	95/560 (17.0)	75/555 (13.5)	81/1082 (7.5)	35/340 (10.3)	522/4041 (12.9)
With ICU admission	9/45 (20.0)	21/76 (27.6)	7/62 (11.3)	12/69 (17.4)	5/132 (3.8)	7/33 (21.2)	61/417 (14.6)
With mech. ventilation	8/33 (24.2)	14/61 (23.0)	7/42 (16.7)	9/40 (22.5)	3/57 (5.3)	2/8 (25.0)	43/241 (17.8)

Columns show: Number of deaths within 30 days of hospitalization/Number of hospitalizations with COVID-19 (%)

Figure S1. Map from the region of Madrid (Comunidad Autónoma de Madrid) with its 179 municipalities. Highlighted in red is the metropolitan area of Madrid.

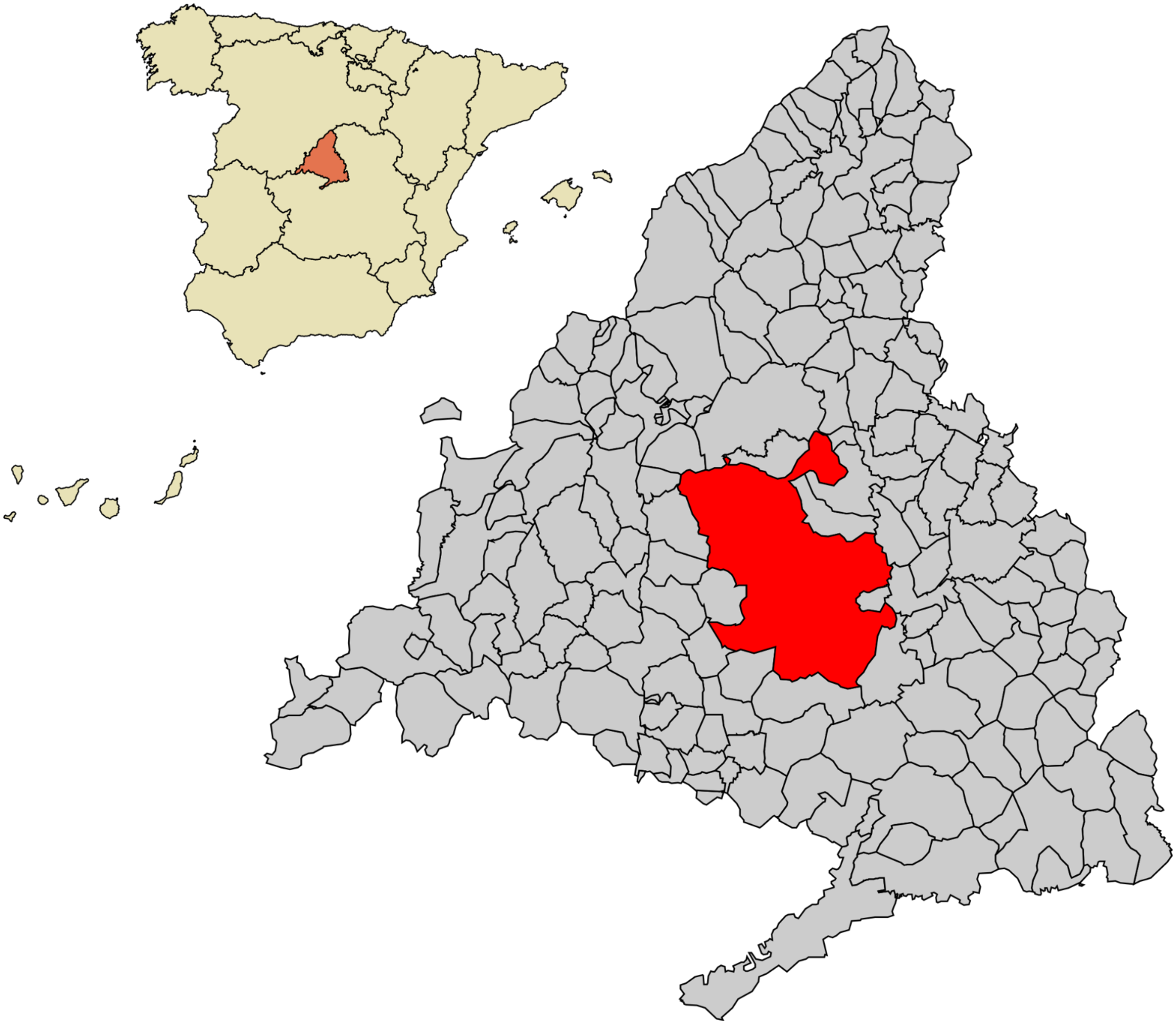

From Wikimedia Commons, the free media repository. https://commons.wikimedia.org/wiki/File:Madrid_%28Comunidad_de_Madrid%29_mapa.svg

Figure S2. Weekly distribution of SARS-CoV-2 variants of concern in the Madrid region, 2021-2022

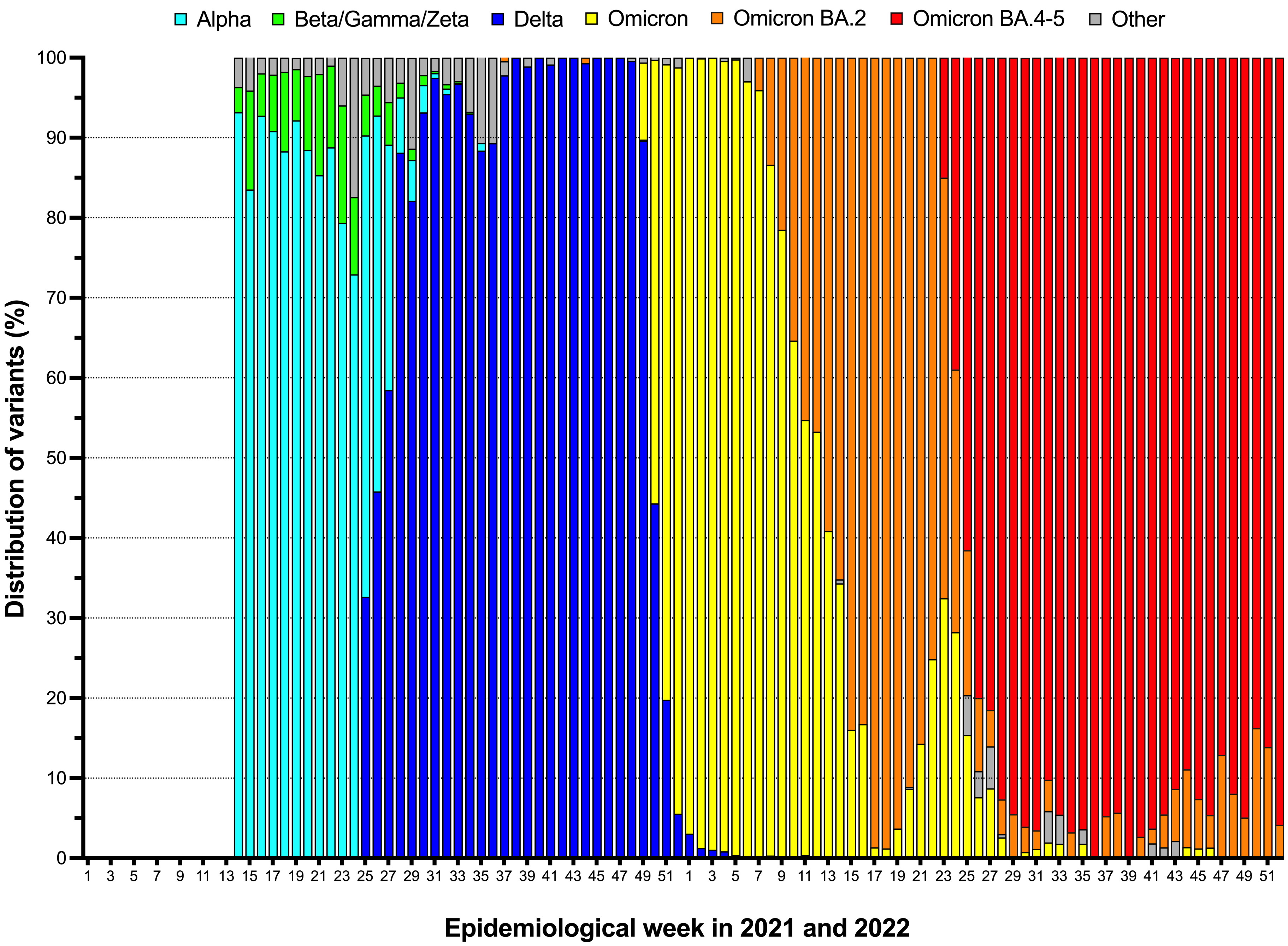

Figure S3. Residents in the Madrid region on December 31, 2019 stratified by age and sex.

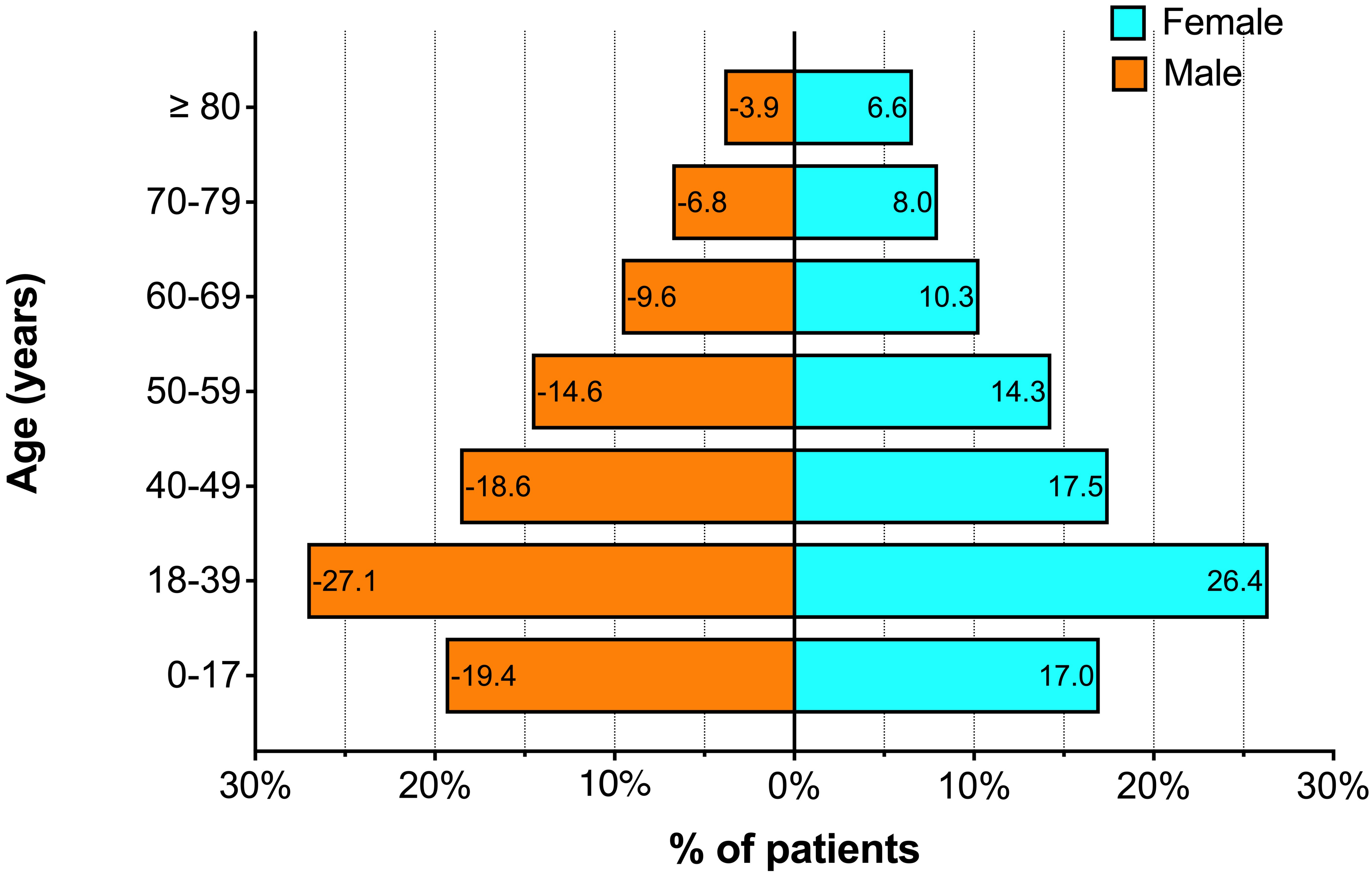

Figure S4. Types of COVID-19 hospitalizations by semesters across the study period, Madrid region, 2020-2022

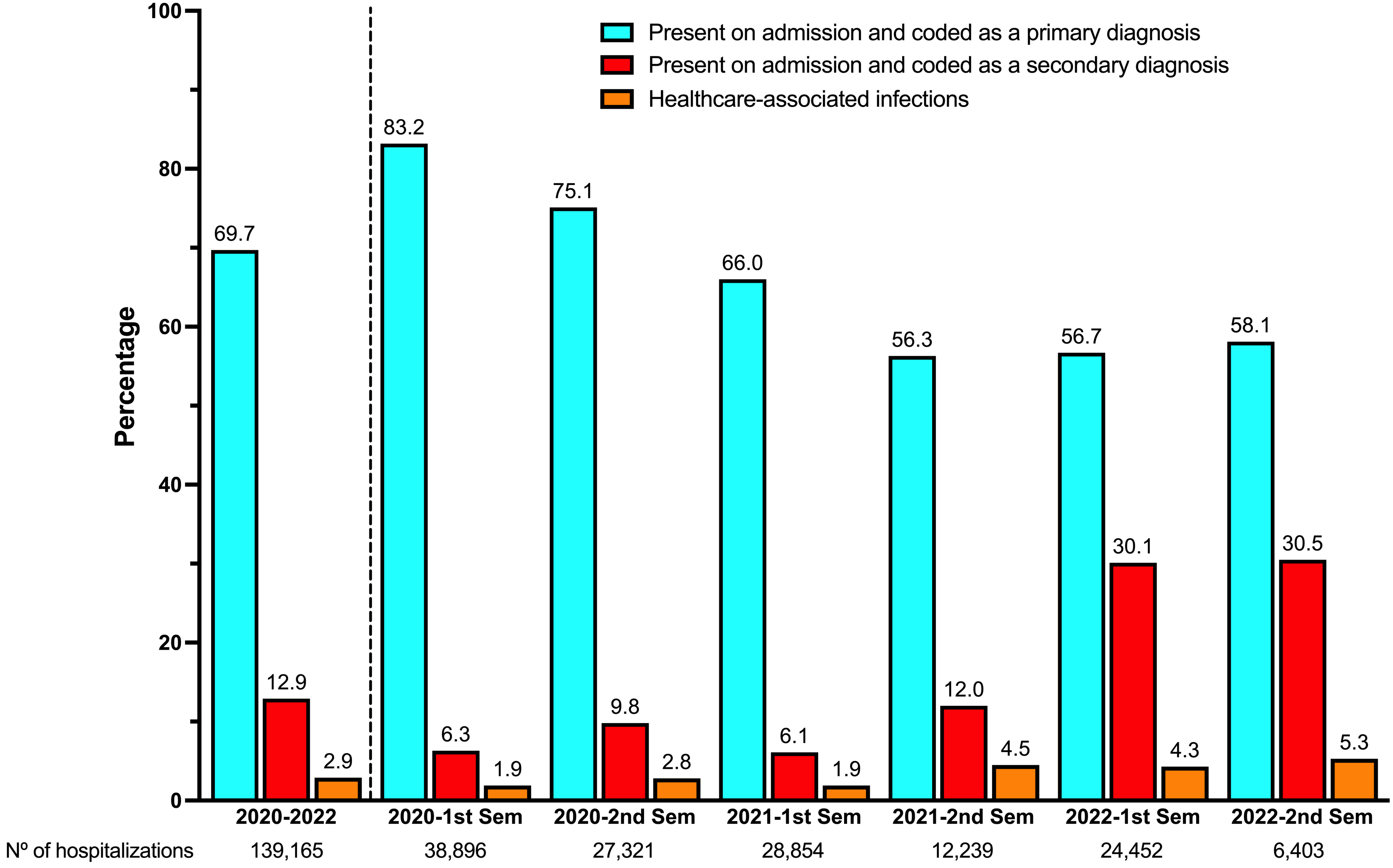

In 14.5% hospitalizations, COVID-19 was not present on admission though criteria for probable or definite hospital-acquired infections were not met.

Figure S5. Cumulative uptake of at least one dose of COVID-19 vaccine overall and by age group, Madrid region, 2021-2022

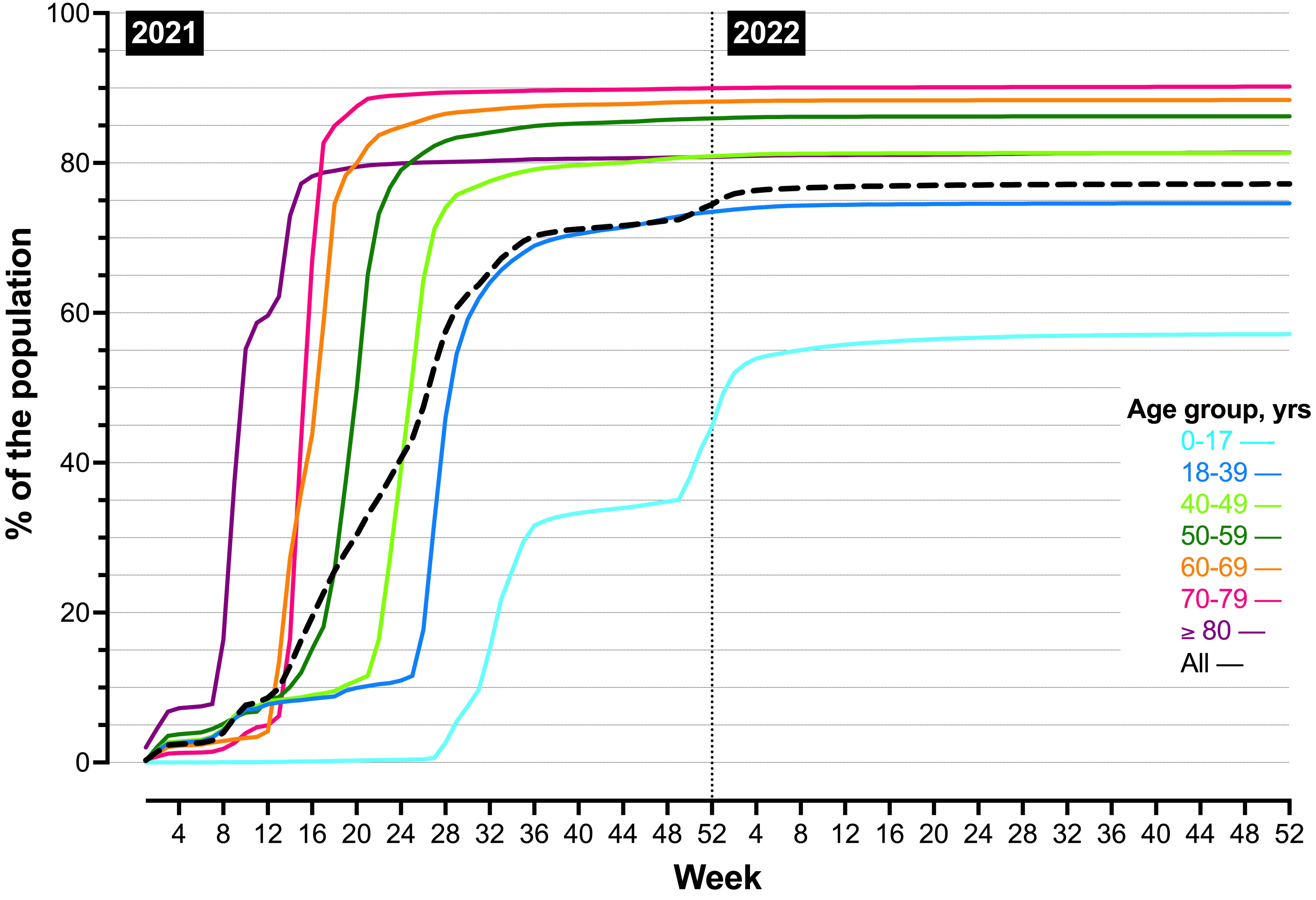

Figure S6. Number of COVID-19 ambulatory infections treated with nirmatrelvir plus Ritonavir and molnupiravir in the Madrid region from April to December 2022

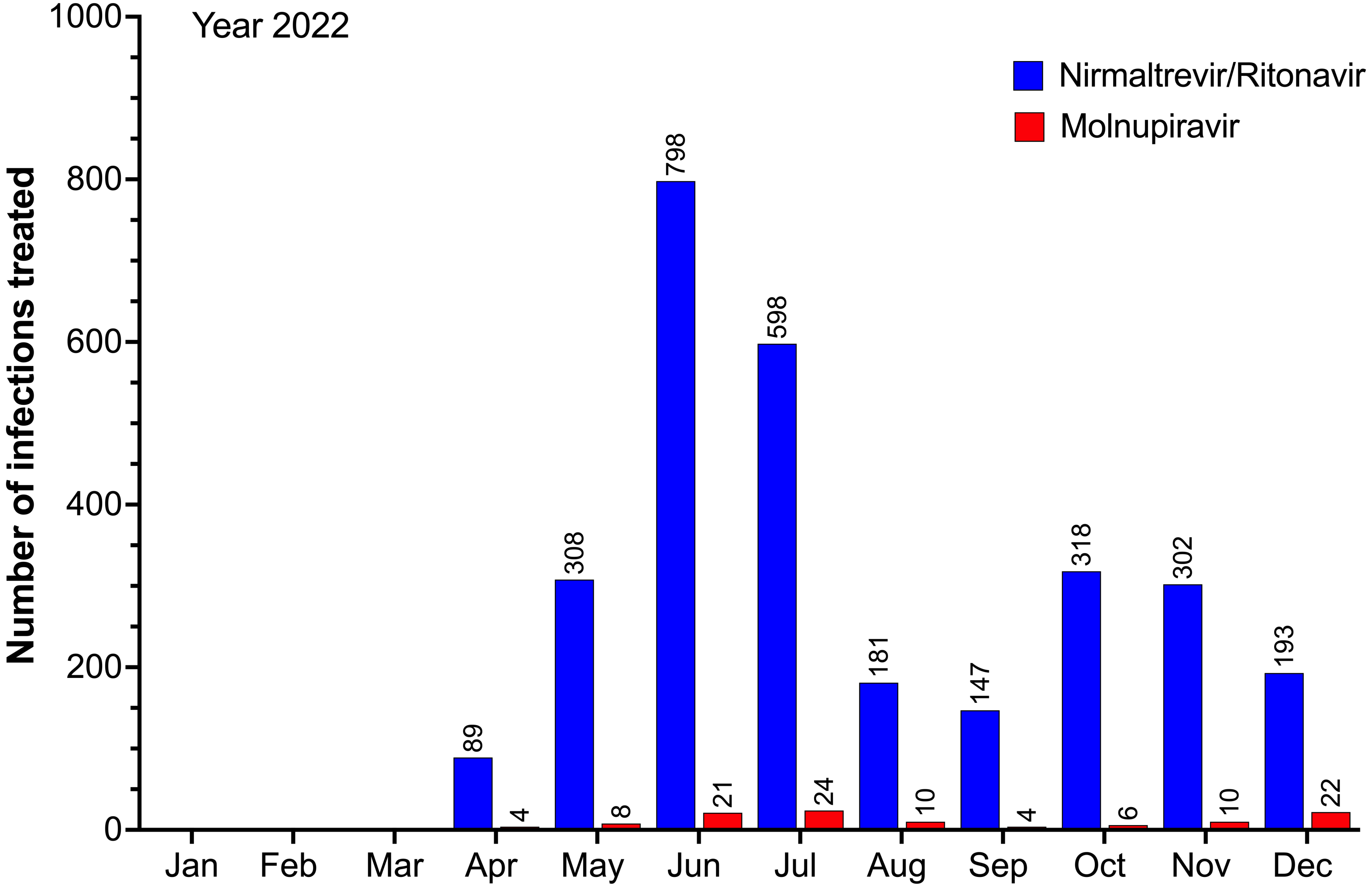

	Jan	Feb	Mar	Apr	May	Jun	Jul	Aug	Sep	Oct	Nov	Dec
Documented Infections	237,313	43,286	21,498	21,793	56,412	70,969	36,390	8,801	6,115	13,388	10,567	6,604

These antivirals were authorized for use within the first five days of symptoms among ambulatory individuals with confirmed COVID-19 and severe immunosuppression or those aged 65 or more with severe comorbidities

Annex 1. International Classification of Primary Care (ICPC-2) Codes of comorbidities registered for this study.

Comorbidity	ICPC-2 Codes
Obesity	T82
Diabetes mellitus	T89, T90
Hypertension	K86, K87
Cardiovascular disease *	K74-K78
Cerebrovascular disease †	K89-K91
Asthma	R96
Chronic obstructive pulmonary disease	R95
Chronic renal failure	U99.01
Connective tissue disease ‡	L88, L88.01, L99
Liver cirrhosis	D97
Solid neoplasms (diagnosed in the previous 5 years)	A79, D74-D77, L71, N74, N76, R84, R85, S77, T71, T73, U75-U77, X75-X77, Y77, Y78, W72
Hematologic neoplasms (diagnosed in the previous 5 years)	B72-B74
HIV infection	B90
Organ transplantation,	A89 (special coding)
Depressive disorder	P76
Psychosis #	P72, P73, P98
Dementia	P70

* Ischemic heart disease, Heart failure, Atrial fibrillation
† Stroke, Transient ischemic attack
‡ Rheumatoid arthritis, Ankylosing spondylitis, Systemic lupus erythematosus, and other connective tissue disorders
# Affective psychosis, Schizophrenia, other organic psychoses

Annex 2. ICD-10-CM Codes

**COVID-19 hospitalization***	Defined by any of the following codes in CMBD [1].<br>1) An ICD-10-CM code B97.29 "other coronavirus as the cause of diseases classified elsewhere" in CMBD before July 1, 2020<br>2) An ICD-10-CM code U07.1 for COVID-19 in CMBD from July 1, 2020
**ICU admission**	Defined by a CMBD code for any critical care unit admission during the hospitalization.
**Mechanical ventilation**	Defined by any of the following ICD-10-CM procedure codes: 5A1935Z, 5A1945Z, 5A1955Z in CMBD.

Abbreviations: ICD-10-CM, International Classification of Diseases, 10th edition, Clinical Modification; CMBD, Spanish Minimum Basic Data Set (Conjunto Mínimo Básico de Datos); ICU, intensive care unit.

1. World Health Organization. Emergency use ICD codes for COVID-19 disease outbreak. Available at: https://www.who.int/standards/classifications/classification-of-diseases/emergency-use-icd-codes-for-covid-19-disease-outbreak.

*Specialists in clinical documentation further characterized COVID-19 hospitalization as whether the infection was present on admission and whether it was the primary or a secondary diagnosis.

- **Primary diagnosis** corresponded to acute or persistently symptomatic infections with potentially life-threatening manifestations (e.g., COVID-19 pneumonia).
- COVID-19 hospitalizations coded as a **secondary diagnosis** include hospitalizations for underlying diseases that are exacerbated or caused by contracting COVID-19 (e.g., heart failure), hospitalizations for unrelated incidental conditions which also happen to have a COVID-19 infection (e.g., bone fracture), and even hospitalizations for disorders for which COVID-19 is believed to be responsible. However, the patient has no active acute illness (e.g., lung embolism).

Annex 3. International Classification of Disease, 10th Revision (ICD-10) Coding Algorithms for Charlson Comorbidities

Comorbidity	ICD-10 Codes
Myocardial infarction	I21.x, I22.x, I25.2
Congestive heart failure	I09.9, I11.0, I13.0, I13.2, I25.5, I42.0, I42.5-I42.9, I43.x, I50.x, P29.0
Peripheral vascular disease	I70.x, I71.x, I73.1, I73.8, I73.9, I77.1, I79.0, I79.2, K55.1, K55.8, K55.9, Z95.8, Z95.9
Cerebrovascular disease	G45.x, G46.x, H34.0, I60.x-I69.x
Dementia	F00.x-F03.x, F05.1, G30.x, G31.1
Chronic pulmonary disease	I27.8, I27.9, J40.x-J47.x, J60.x-J67.x, J68.4, J70.1, J70.3
Rheumatic disease	M05.x, M06.x, M31.5, M32.x-M34.x, M35.1, M35.3, M36.0
Peptic ulcer	K25.x-K28.x
Diabetes without chronic complication	E10.0, E10.1, E10.6, E10.8, E10.9, E11.0, E11.1, E11.6, E11.8, E11.9, E12.0, E12.1, E12.6, E12.8, E12.9, E13.0, E13.1, E13.6, E13.8, E13.9, E14.0, E14.1, E14.6, E14.8, E14.9
Diabetes with chronic complication	E10.2-E10.5, E10.7, E11.2-E11.5, E11.7, E12.2-E12.5, E12.7, E13.2-E13.5, E13.7, E14.2-E14.5, E14.7
Hemiplegia or paraplegia	G04.1, G11.4, G80.1, G80.2, G81.x, G82.x, G83.0-G83.4, G83.9
Renal disease	I12.0, I13.1, N03.2-N03.7, N05.2-N05.7, N18.x, N19.x, N25.0, Z49.0-Z49.2, Z94.0, Z99.2
Liver disease, mild	B18.x, K70.0-K70.3, K70.9, K71.3-K71.5, K71.7, K73.x, K74.x, K76.0, K76.2-K76.4, K76.8, K76.9, Z94.4
Liver disease, moderate or severe	I85.0, I85.9, I86.4, I98.2, K70.4, K71.1, K72.1, K72.9, K76.5, K76.6, K76.7
Any malignancy*	C00.x-C26.x, C30.x-C34.x, C37.x-C41.x, C43.x, C45.x-C58.x, C60.x-C76.x, C81.x-C85.x, C88.x, C90.x-C97.x
Metastatic solid tumor	C77.x-C80.x
HIV/AIDS	B20.x-B22.x, B24.x

*Including lymphoma and leukemia, except malignant neoplasm of skin

Reference: Quan H, Sundararajan V, Halfon P, et al. Coding algorithms for defining comorbidities in ICD-9-CM and ICD-10 administrative data. *Med Care*. 2005;43(11):1130-1139.
